# Application and evaluation of Flipped Teaching based on Video Conference in standardized training for internal medicine residents

**DOI:** 10.1371/journal.pgph.0001874

**Published:** 2023-05-09

**Authors:** Xiao-Yu Zhang

**Affiliations:** 1 Section of Education, Shanghai Public Health Clinical Center, Fudan University, Shanghai, P. R. China; 2 Department of Liver Disease, Shanghai Public Health Clinical Center, Fudan University, Shanghai, P. R. China; 3 Public Health Education Professional Committee, Shanghai Preventive Medicine Association, Shanghai, P.R. China; PLOS: Public Library of Science, UNITED STATES

## Abstract

**Background:**

In view of the importance of infectious diseases in public health, Shanghai Municipal Health Commission designated a hospital (“Designated Hospital”) to carry out infectious diseases training for internal medicine residents in those hospitals (“Dispatching Hospitals”) that didn’t have an infectious disease ward or could not meet the training standards of infectious diseases.

**Objective:**

I aimed to explore Flipped Teaching with Video Conference as the carrier in infectious diseases training for internal medicine residents, to make up for the lack of actual training time of the Department of Infectious Diseases for those residents caused by subjective or objective reasons, and to ensure the smooth implementation and quality assurance of infectious diseases training for those residents.

**Methods:**

Vertical management mode was adopted, management and lecture teams were established, and training program and teaching implementation were formulated. Flipped Teaching based on Video Conference was carried out for internal medicine residents of Dispatching Hospitals who planned to participate in infectious diseases training of the Designated Hospital in April. The quantitative analysis was applied to this teaching evaluation, and the evaluation indexes were included into statistical analysis to evaluate the effect of the teaching model.

**Results:**

All 19-member internal medicine residents participated in the Flipped Teaching based on Video Conference from April 1 to 4, of which 12 residents were scheduled to participate in infectious diseases training from March 1 to April 30, and 7 residents were scheduled to participate in infectious diseases training from April 1 to May 31 in the Designated Hospital. A management team of 6 internal medicine residents was built, and a lecture team was composed of 12 internal medicine residents who were scheduled to receive infectious diseases training in the Designated Hospital from March 1 to April 30. According to the training requirements in the Department of Infectious Diseases, 12 contents were selected to be taught, and implementation rate of the teaching plan was over 90%. A total of 197 feedback questionnaires were collected. The feedback that the teaching quality was "good" and "very good" accounted for more than 96%, and the attendance rate of the whole teaching process reached more than 94%. Six internal medicine residents put forward 18 "Improvement suggestions", accounting for 9.1%; and 11 internal medicine residents gave 110 suggestions of "Praise highlights", accounting for 55.8%. The overall evaluation feedback of Flipped Teaching was good, P<0.001.

**Conclusion:**

Flipped Teaching based on Video Conference was generally effective in delivering lectures and learning for internal medicine residents participating in the infectious diseases training, and it could be used as a supplementary training method for standardized training of internal medicine residents to make up for the shortage of actual training period in a certain stage.

## Introduction

In view of the rapid evolution of the disease spectrum of infectious diseases in Shanghai in recent years and the need to effectively response to public health emergencies, the Department of Infectious Diseases was chosen to be included in the compulsory rotation discipline of standardized training for internal medicine residents, so as to further improve the knowledge structure of internal medicine residents and meet the needs of society for the diagnosis and treatment of infectious diseases. For training hospitals without infectious disease wards or not meet the requirements for the training of the Department of Infectious Diseases, their internal medicine residents should be arranged to participate in the infectious diseases training of the Designated Hospital [1].

With the continuous promotion of the infectious diseases training for internal medicine residents in Shanghai, more and more hospitals participated in the collaborative training of internal medicine residents in the Designated Hospital, and the number of internal medicine residents joining the infectious diseases training of the Designated Hospital gradually had been increasing. According to the requirements of the standardized training program for internal medicine residents, those residents attending the infectious diseases training in the Designated Hospital needed to carry out professional training for 1–2 months. In order to unify management and training, a certain number of internal medicine residents would join the Designated Hospital for infectious diseases training every month, and should be connected in an orderly manner to ensure the quality of training and rational utilization of public medical resources. During the operation of the project, the training cycle of internal medicine residents in the Designated Hospital for infectious diseases training might be insufficient, due to holidays or vacations, coordination of training dates between multiple hospitals, and other subjective and objective reasons. Therefore, I explored a new teaching mode to further improve the infectious diseases training for internal medicine residents.

In the Flipped Teaching research report, the flipped classroom approach in health professions education yielded a significant improvement in student learning compared with traditional teaching methods [2], the flipped classroom and lecture were essentially equivalent [3], and an increased perceived value and acceptability of this model was noted by the participants [4]. Therefore, I explored the use of Video Conference as the carrier to carry out Flipped Teaching, and constructed the teaching mode of "Flipped Teaching in Standardized Training for Internal Medicine Residents Based on Video Conference" to perform distance online teaching. The mode was applied to the infectious diseases training for internal medicine residents, and preliminary evaluation was conducted.

## Methodology

### Study population

The study population were internal medicine residents who planned to take training in the Designated Hospital in April. And the target population were the residents who had participated in the standardized training for residents in Shanghai. The Dispatching Hospitals had signed an "Agreement on Joint Training of Internal Medicine Residents" with the Designated Hospital.

The verbal informed consents of participants in internal medicine residents were obtained, including their data being used for the training and the research, and that this study was conducted in accordance with the Declaration of Helsinki. The study received Institutional Review Board (IRB) approval by the Shanghai Public Health Clinical Center Ethics Committee. The IRB number was No. 2021-S026-01.

### Construction of Flipping Teaching mode with Video Conference as the method

The Flipped Teaching with Video Conference as the method adopted "vertical management mode" for management [5], and management and lecture teams were established. According to the training requirements of the Department of Infectious Diseases in the Standardized Training Content and Standard for Resident Physicians (2021 Edition)—Internal Medicine Training Rules, the management team would formulate the training program, and the lecture team would select the training content and carry out teaching activities according to the plan. The following was the implementation and evaluation of the teaching plan and work respectively. The whole teaching organization, implementation, management, discussion and evaluation were carried out online without restriction of physical space.

### Evaluation indexes and criteria

This teaching model was evaluated from four aspects, including the implementation of the teaching plan, the attendance of the Flipped Teaching, the evaluation of teaching quality, and the overall evaluation of teaching.

Six teaching plan indicators (teaching on the planned time, teaching on the planned content, making PPT fully, providing references, unifying the teaching content and training program, and participating in after-class discussion) were established to evaluate the implementation of the teaching plan. Three attendance indicators (online on time, middle roll call and end on time) were established to evaluate the online attendance of the Flipped Teaching. Nine teaching quality indicators (rigorous teaching attitude, punctual class, detailed and accurate teaching content, reasonable structure and clear process, highlighting teaching key points, clear teaching difficulties, accurate and refined language, combining theory with clinical practice, improving ability to analyze and deal with the disease) were established to evaluate the teaching quality. The overall evaluation of teaching adopted open questionnaire, and carried on the unlimited evaluation to each teaching item.

The teaching plan indicators and the attendance indicators were completed by the organizer, and the teaching quality indicators and the overall evaluation content were completed by every trainee for each teaching session. Among them, teaching plan indicators and teaching quality indicators were objective indicators, and they were derived from Teaching Evaluation Table of Standardized Residency Training; while overall evaluation indicators were subjective. The feedback of the over evaluation from open questionnaire was firstly classified according to the evaluation content. The details of classification were as follows: If the content of a questionnaire feedback was pointing out deficiencies or needing improvement of one teaching session, this feedback was classified as "Improvement suggestions"; If all the content of a questionnaire feedback was praising highlights or learning achievements of one teaching session, this feedback was classified as "Praise highlights"; If the content of a questionnaire feedback was no special suggestions of one teaching session, this feedback was classified as "No special suggestions".

### Software application and statistical analysis

Video Conference adopted Tencent Conference software to carry out the Flipped Teaching, including: teaching organization, implementation, discussion after teaching, management and evaluation.

The Questionnaire Star software was used to develop teaching quality indicators and overall evaluation indicators, and carry out star survey after class; and then the data of above indicators were downloaded from the software and incorporated into statistical analysis.

SPSS software version 23.0 (SPSS Inc. Chicago, IL, USA) was used for statistical analysis of the data. The data conforming to normal distribution were expressed as mean ± standard deviation to reflect the distribution of the study indicators. The counting data was represented by example (%) to reflect the composition ratio of the study indicators. Pearson chi-square test was used for the counting data. A P value of two-sided less than 0.05 was considered as statistically significant.

## Results

### Basic information of internal medicine residents participating in teaching activities

A total of 19 internal medicine residents participated in the Flipped Teaching program, all from tertiary hospitals. Among them, 9 were male, accounting for 47.4%. The average age was 29.5 years. Five had a bachelor’s degree, accounting for 26.3%; 4 had a master’s degree, accounting for 21.1%; and 10 had a doctoral degree, accounting for 52.6%. Seventeen were qualified as practicing physicians, accounting for 89.5%. Two were in the first year of training, accounting for 10.5%; 12 were in the second year of training, accounting for 63.2%; and 5 were in the third year of training, accounting for 26.3%. Twelve were scheduled to participate in infectious diseases training in the Designated Hospital from March 1 to April 30, accounting for 63.1%; and 7 were scheduled to participate in infectious diseases training in the Designated Hospital from April 1 to May 31, accounting for 36.8%. The detailed information is shown in **Table 1**.

**Table 1:** Baseline of internal medicine residents participating in Flipped Teaching.

Indicators		
Total number [n,(%)]		19(100)
Sexuality [male,n,(%)]		9(47.4)
Age [years,Mean±standard deviation]		29.5±3.1
Education background		
	Bachelor’s degree[n,(%)]	5(26.3)
	Master’s degree[n,(%)]	4(21.1)
	Doctoral degree[n,(%)]	10(52.6)
Qualified as a licensed physician [n,(%)]		17(89.5)
Training phase		
	First year of training[n,(%)]	2(10.5)
	Second year of training[n,(%)]	12(63.2)
	Third year of training[n,(%)]	5(26.3)
From tertiary hospitals [n,(%)]		19(100)
Training period for infectious diseases training in the Designated Hospital as planned		
	March 1—April 30 [n,(%)]	12(63.1)
	April 1—May 31 [n,(%)]	7(36.8)

### Flipped Teaching organization

The Flipped Teaching with Video Conference as the carrier was organized and implemented by the contact person of the "Rotation Training of Infectious Diseases Department for Internal Medicine Residents" project, which was managed by the "vertical management mode". The management team was formed together with the monitor and group leaders of this training course, and 12 lecturers were composed of those residents who were scheduled to take part in the infectious diseases training of the Designated Hospital from March 1 to April 30.

The contents of the lecture were as follows: Analysis of Chronic Hepatitis B, Study on Guidelines for Prevention and Treatment of Hepatitis C (2019 Edition), Identification and Treatment of Clostridium Difficile Associated Diarrhea, Bacterial Liver Abscess, Diagnosis and Treatment of Tuberculosis, Guidelines for the Diagnosis and Treatment of Syphilis, Diagnosis and Treatment of Tuberculous Meningitis, Infective Endocarditis, Cryptococcal Meningitis, Study on AIDS Diagnosis and Treatment Guide in China (2021 edition), Diagnosis and Treatment of Cirrhotic Ascites, and Diagnosis and Treatment of liver Failure. Teaching tasks were assigned to those residents according to the time period, and PPT was developed to carry out teaching activities according to clinical guidelines. The contents of the lecture are shown in **Table 2**.

**Table 2:** Flipped Teaching plan.

Teaching date	Teaching time	content of courses
April 1	13:35–14:20	Analysis of Chronic Hepatitis B
April 1	14:35–15:20	Study on Guidelines for Prevention and Treatment of Hepatitis C (2019 Edition)
April 1	15:35–16:20	Identification and Treatment of Clostridium Difficile Associated Diarrhea
April 2	13:35–14:20	Bacterial Liver Abscess
April 2	14:35–15:20	Diagnosis and Treatment of Tuberculosis
April 2	15:35–16:20	Guidelines for the Diagnosis and Treatment of Syphilis
April 3	13:35–14:20	Diagnosis and Treatment of Tuberculous Meningitis
April 3	14:35–15:20	Infective Endocarditis
April 3	15:35–16:20	Cryptococcal Meningitis
April 4	13:35–14:20	Study on AIDS Diagnosis and Treatment Guide in China (2021 edition)
April 4	14:35–15:20	Diagnosis and Treatment of Cirrhotic Ascites
April 4	15:35–16:20	Diagnosis and Treatment of liver Failure

### Evaluation of teaching plan implementation

The Flipped Teaching was carried out according to plan, with real-time online management and evaluation. In the teaching process, one of the lecturers delayed the start time of teaching on the planned time, because he was not familiar with Video Conferencing software; and the rest of the lecturers carried out teaching activities on time. Teaching plan met the requirements for 11 times, accounting for 91.7%. All the other teaching plan indicators were in compliance with the coincidence rate of 100%. The detailed information is shown in **Table 3**.

**Table 3:** Evaluation of teaching plan implementation.

Teaching plan indicators	CT	NCT	CR (%)
Teaching on the planned time	11	1	91.7
Teaching on the planned content	12	0	100
Making PPT fully	12	0	100
Providing references	12	0	100
Unifying the teaching content and training program	12	0	100
Participating in after-class discussion	12	0	100

**Abbreviations**: CT, Coincidence times; NCT, Not-Coincidence times; CR, Coincidence rate.

**Note**: Coincidence rate (%) = Coincidence times of teaching indicators / Total times of teaching indicators *100%.

### Teaching attendance and feedback

The whole process of attendance was checked for this Flipped Teaching based on Video Conference, and the three time nodes of "online on time", "middle roll call" and "end on time" were included in the statistics. There were 18, 18 and 19 internal medicine residents in attendance at the above three time nodes, and the attendance rates were 94.7%, 94.7% and 100%, respectively. One of those residents failed to go online on time because he was not familiar with Video Conference software, and one asked for leave and went offline due to an emergency. The detailed attendance is shown in **Table 4**.

**Table 4:** Attendance of Flipped Teaching.

Attendance indicators	Residents in attendance	Attendance rates(%)
Online on time	18	94.7
Middle roll call	18	94.7
End on time	19	100

**Note**: Attendance rate (%) = Number of internal medicine residents in attendance / Total number of internal medicine residents of this Flipped Teaching for the infectious training *100%

### Quality evaluation of teaching

The teaching quality of the Flipped Teaching based on Video Conference was investigated from the questionnaire star, and 179 effective feedback questionnaires were collected. Feedback "good" of nine indicators ("Rigorous teaching attitude", "Punctual class", "Detailed and accurate teaching content", "Reasonable structure and clear process", "Highlighting teaching key points", "Clear teaching difficulties", "Accurate and refined language", "Combining theory with clinical practice", and "Improving ability to analyze and deal with the disease") accounted for 13.2%, 14.7%, 18.3%, 16.8%, 16.3%, 16.8%, 15.8%, 14.3% and 15.3%, respectively. And Feedback "very good" of the above nine indicators accounted for 86.3%, 84.8%, 79.7%, 80.7%, 82.2%, 79.7%, 81.7%, 82.7% and 82.7%, respectively. On the whole, teaching quality was rated as "good" and "very good" by more than 96%. The detailed evaluation of teaching quality is shown in **Table 5**.

**Table 5:** Quality evaluation of Flipped Teaching.

Teaching quality indicators[n,(%)]	VP(%)	PR(%)	GN(%)	GD(%)	VG(%)
Rigorous teaching attitude	0(0)	0(0)	1(0.5)	26(13.2)	170(86.3)
Punctual class	0(0)	0(0)	1(0.5)	29(14.7)	167(84.8)
Detailed and accurate teaching content	0(0)	0(0)	4(2.0)	36(18.3)	157(79.7)
Reasonable structure and clear process	0(0)	0(0)	5(2.5)	33(16.8)	159(80.7)
Highlighting teaching key points	0(0)	0(0)	3(1.5)	32(16.3)	162(82.2)
Clear teaching difficulties	0(0)	0(0)	7(3.5)	33(16.8)	157(79.7)
Accurate and refined language	0(0)	0(0)	5(2.5)	31(15.8)	161(81.7)
Combining theory with clinical practice	0(0)	0(0)	6(3.0)	28(14.3)	163(82.7)
Improving ability to analyze and deal with the disease	0(0)	0(0)	4(2.0)	30(15.3)	163(82.7)

**Abbreviations**: VP, Very poor; PR, Poor; GR, General; GD, Good; VG, Very good.

**Note**: 197 feedback of teaching quality evaluation were collected through questionnaires.

### Overall evaluation of teaching

In the overall evaluation of the Flipped Teaching based on Video Conference, 6 internal medicine residents filled in "Improvement suggestions" feedback in 18 questionnaires, accounting for 9.1%; 11 internal medicine residents proposed "Praise highlights" feedback in 110 questionnaires, accounting for 55.8%; 10 internal medicine residents had "No special suggestions" feedback in 69 questionnaires, accounting for 35.0%. The overall evaluation feedback of Flipped Teaching based on Video Conference was good, P<0.001. The detailed overall evaluation of feedback is shown in **Table 6**.

**Table 6:** Overall evaluation of Flipped Teaching.

Evaluation indicators	NPE	RPE, CI (%)	P	TTE	RTET, CI (%)	P
Improvement suggestions	6	31.6(8.6–54.6)	0.228	18	9.1(5.1–13.2)	<0.001
No special suggestions	10	52.6(22.9–77.4)		69	35.0(28.3–41.7)	
Praise highlights	11	57.9(33.4–82.3)		110	55.8(48.8–62.8)	

**Abbreviations**: NPE, Number of participants in evaluation; RPE, Ratio of participants in evaluation; TTE, Times of teaching evaluations; RTET, Ratio of teaching evaluation time; CI, 95% confidence interval; P, P value.

**Note**: Ratio of participants in evaluation (%) = Number of participants in evaluation / Total number of participants in the infectious diseases training *100%

Ratio of teaching evaluation times (%) = Teaching evaluation times of each indicator of overall evaluation / Total teaching evaluations times of overall evaluation *100%

197 feedback of teaching evaluations on overall evaluation were collected through the questionnaires.

## Discussion

The participation of internal medicine residents in the infectious diseases training was carried out to perfect the knowledge structure of the standardized training of internal medicine residents and the social needs for the diagnosis and treatment of infectious diseases. Those training hospitals that did not meet the training standards for infectious diseases should cooperate with the Designated Hospital to conduct infectious diseases training for internal medicine residents. Due to the fact that the internal medicine residents who participated in the infectious diseases training of the Designated Hospital came from more than 10 hospitals in Shanghai, the personnel management, implementation plan and training progress of internal medicine residents in each hospital were different to some extent. In order to ensure the quality of internal medicine residents’ training and rationally allocate the training resources, a certain number of internal medicine residents were allowed to participate in the infectious diseases training at the Designated Hospital every month. In the actual operation, some internal medicine residents scheduled to take part in the infectious diseases training in the Designated Hospital could not attend the training on time or meet the training time requirements due to various reasons. To this end, I applied Flipped Teaching based on Video Conference to carry out infectious diseases training for internal medicine residents. Therefore, it was obviously necessary to carry out the application and research on this teaching model.

Flipped classrooms showed many advantages when tested in a radiology classroom setting, making up for some inadequacies of didactic classrooms; but there was a need to make improvements so that it’s more suitable for the Chinese medical education mode [6]. The flipped classroom approach showed promise in ophthalmology clerkship teaching; but, it had some drawbacks; further evaluation and modifications were required before it could be widely accepted and implemented [7]. Based on the above, I carried out this teaching model research. The whole Flipped Teaching mode involved organizational structure, training content, plan, implementation, management and evaluation. This teaching research was a prospective study, establishing organizational structure and management model, developing training content and plan. The implementation and quality analysis of the teaching model was evaluated by assessment and after-class questionnaire survey. Then the practicability, rationality, feasibility and effectiveness of this teaching model were evaluated. Therefore, this teaching research had distinct scientific nature.

The teaching organization structure included training organization, management model, management and lecture teams. This training was organized and implemented by the contact person of the "Rotation Training of Infectious Diseases Department for Internal Medicine Residents" project, to ensure the cooperation and support of teaching administrative departments and internal medicine residents of each Dispatching Hospital, and to mobilize the enthusiasm of internal medicine residents for this infectious diseases training project. A management team of six was set up in this teaching work, which were used to assist the project contact person to organize and implement this teaching activity, and supervised the teaching plan and supporting work, in order to further develop those residents’ subjective initiative. A total of 12 lecturers were organized, consisting of those residents who planned to attend training on the infectious diseases in the Designated Hospital from March 1 to April 30. As a result, they had one month of clinical training experience in the Department of Infectious Diseases. They chose target diseases to give lectures based on their own clinical practice and shared their learning experience of this disease. In this way, the teaching could encourage the lecturers to learn and summarize the clinical knowledge of the selected diseases. Twelve lecturers were selected in this teaching to encourage more qualified lecturers to participate in teaching activities and ensure the coverage of the teaching content. This teaching adopted vertical management mode. Since good experience and evaluation was gained in the "vertical management" mode in the field of standardized training for public health physicians [5], it was also adopted in this teaching management and achieved obvious results, which reflected that the teaching model had good practicability.

The content of this teaching was based on the establishment of the training program of the syllabus for internal medicine residents, with the orientation of the essential and key diseases in the infectious diseases training. The goal of this teaching was to train internal medicine residents on the diagnosis and treatment skills of infectious diseases. The main contents were based on the clinical diagnosis and treatment guidelines of infectious diseases. All the above was to ensure that the teaching purpose was consistent with requirements of the infectious diseases training, and the standardized training of internal medicine residents. So, 12 diseases were selected as the main topics of the teaching, covering all the diseases that must be mastered, and involving other key diseases of the infectious diseases training. Therefore, the teaching and training content was reasonably designed, which met the needs of the infectious diseases training at the level of internal medicine residents and reflected the rationality of the teaching model.

In the part of planning implementation, it was an important part of teaching work to formulate training plans and organize implementation under the condition of definite training content. In order to carry out the teaching contents in an orderly manner, targeted teaching plans were drawn up, and the teaching contents needed to be implemented for specific personnel and definite time periods, so that the lecturers and participating residents could make full preparations. The management group made a good teaching time frame; while the lecturers took the initiative to participate, and selected their own lecturing content and time. Once the content of the lecture was determined, no modification would be made unless in an emergency situation. If the teaching could not be carried out within the planned time, the lecturer’s teaching would be adjusted to the last time period in an orderly manner. The original plan of the 12 disease teaching tasks was to be completed in 4 days, and three time periods were arranged for orderly teaching activities every afternoon. In the implementation of the teaching plan, it was adopted for unified planning, group management, classified hosting, punctual teaching, online discussion, whole attendance, and "Questionnaire Star" survey feedback after class. In this teaching activity, one of the lecturers delayed the start time of teaching, because he was not familiar with Video Conference software. Therefore, in the formal organization of teaching activities, teaching preparation should be done more carefully for every section. For example, software drills and trial lectures should be carried out in the early stage of teaching. In the process of teaching implementation, the proportion of "Teaching on the planned time" was 91.7%, and the proportion of "Teaching on the planned content", "PPT making fully", "Providing references", "Unifying teaching content and training outline" and "Participation in after-class questions" were all 100%. From the perspective of teaching arrangement and implementation, this teaching plan had been implemented smoothly and had strong feasibility.

This Flipped Teaching based on Video Conference adopted online whole-process monitoring, and three time nodes of "On-time online", "Middle roll call" and "On-time end" were included in the statistics. The attendance rate was 94.7%, 94.7% and 100%, respectively. One of them failed to go online on time because he was not familiar with the Video Conference software, and one of them asked for leave and went offline because of emergency during the process. From the statistics of the above three time nodes, whole-process monitoring was helpful to stabilize the teaching attendance rate. It was also conducive for mastering the teaching of emergency, timely discovery, and timely treatment. From the perspective of teaching management experience, online questioning could motivate the lecturers to prepare for teaching, promote the lecturers to take the initiative to learn for knowledge reserve, and mobilize the active learning consciousness and enthusiasm of those residents. And the attendance of this teaching model was higher than that of other types of flipped classes, where attendance was 30–80% [8]. Through the Questionnaire Star to the teaching quality of 9 indicators feedback, the results showed that the teaching quality was "Good" and "Very good" accounting for more than 96%. In other specialized clinical training, flipped classroom was also well received and preferred, and it improved teaching satisfaction [9–11]. In terms of the attendance and teaching quality evaluation of internal medicine residents, the effectiveness of this teaching model was relatively good.

In the overall evaluation of this teaching, 11 internal medicine residents raised "Praise highlights" in 110 questionnaires, accounting for 55.8%; which was higher than other feedback, P<0.05. It reflected that the Flipped Teaching based on Video Conference was generally recognized, and the opinion was consistent with other clinical training studies. Røe Y et al suggested that a flipped classroom approach in physiotherapy education resulted in improved student performances in this professional programme [12]. Graham KL suggested flipped classroom for cardiovascular prevention curriculum showed greater effectiveness in knowledge gain [13]. However, 10 internal medicine residents had "No special suggestions" feedback among the 69 questionnaires; and 6 internal medicine residents filled in "Improvement suggestions" feedback in 18 questionnaires, accounting for 9.1%. The above feedback also suggested that the teaching mode still needed to be improved. In promoting the application of this mode, the organizer also needed to follow up the training work in real time, collected the feedback of those residents, and made continuous improvement based on the requirements of infectious diseases discipline in the standardized training for internal medicine residents.

## Conclusions and perspectives

The Flipped Teaching with Video Conference method for internal medicine residents participating in the infectious diseases training was generally effective. The degree of participation and recognition of those residents in this teaching model was relatively good, and the implementation of teaching program was feasible. Application of this teaching mode could make up for the shortage of actual training time of residents in a certain stage.
